# Writing the dark matter of the human genome into mice to better replicate human disease

**DOI:** 10.1093/synbio/ysae003

**Published:** 2024-01-16

**Authors:** David M Truong

**Affiliations:** Department of Biomedical Engineering, NYU Tandon School of Engineering, Brooklyn, NY 11201, USA; Department of Pathology, NYU Langone Health, New York, NY 10016, USA

Since revealing the 3.2 billion nucleotide human genome sequence, researchers have speculated on the function of the 99% of the genome that does not code for protein—which some refer to as the ‘dark matter’ of the genome. These vast stretches of DNA hide the regulatory ‘codes’ for telling genes when, where and by how much to be expressed in every cell type in the body. We still do not fully understand this code, which notably contains ∼90% of human disease-associated DNA variations. Now, Zhang et al. (1), working in Jef Boeke’s group at New York University Grossman School of Medicine in the USA, developed a way to study these human regulatory codes by genetically ‘writing’ as much as 180 kilobases of human DNA in place of the corresponding mouse regions in mouse embryonic stem (ES) cells.

This is an exciting development for those using mice as models for human disease. For many diseases, the mouse genes do not accurately replicate the phenotypes found in humans. Some genes do not even exist. While mice engineered with human genes have been used for decades, these models are limited by the current precision genome editing size limits of around 10 kilobases. More importantly, these models are limited to using the mouse regulatory code for gene expression, which often fails to reflect human biology.

Regulatory DNA comprises the gene promoter along with randomly scattered bits of sequence called enhancers hidden across huge swathes of DNA anywhere from 5 to 100s of kilobases away from the gene. These sequences and patterns differ greatly between humans and mice, resulting in genes being expressed in different cell types and at different levels. The new technique, called ‘mSwap-In’, enables the introduction of nearly 20 times more DNA in a site-specific location compared to previous methods, enough to include all the human codes with its genes into mice.

The researchers demonstrated the scope and use of their method by engineering a human ACE2 mouse model that better modeled human COVID-19 infection patterns. The newly developed method, mSwap-In, thereby uses a combination of CRISPR/Cas9, synthetic DNA assembly and antibiotic markers to write the 180 kilobase human *ACE2* region in place of the comparatively smaller 72 kilobase region into the ES cells of mice. These engineered mouse ES cells were then rapidly turned into full-grown mice using a method called tetraploid complementation.

The *ACE2* gene makes the key protein found on cells allowing the corona virus SARS-CoV-2 to enter the cell. Mice are naturally resistant to SARS-CoV-2; whereas a previously made engineered human ACE2 mouse model expressing too much ACE2 protein on the cell surface resulted in the mice dying too fast from COVID (2). In contrast, the newly engineered mouse model had mild infection symptoms more consistent with those observed in humans (1). Moreover, the cell and tissue expression patterns more accurately reflected those found in humans, with ACE2 now found in the mouse testis and expressed at lower levels in the lung. This is consistent with the differences in the regulatory code.

Mouse models like these may help reduce the failure rate of therapeutics, which are often tested first in normal mice. It also ushers in the era of mammalian genome writing. The next challenge is genetic writing at this scale in human cells. The authors state that mSwap-In would likely work for other species. It should be noted that human pluripotent stem cells (hPSCs) die more easily in response to CRISPR/Cas9 editing (3). A similar method used in hPSCs by the Aizawa group in Japan only identified three cell clones with a 10 kilobase integration (4). Given the cellular differences, it will be interesting to know whether it’s possible to do the same for human stem cells.
